# Supplementary material for: Full-annual demography and seasonal cycles in a resident vertebrate
Source: PeerJ. 2020 Feb 25;8:e8658. doi: 10.7717/peerj.8658 (PMC7047866; doi:10.7717/peerj.8658)
Supplement: Appendix S1 [file peerj-08-8658-s001.pdf]

Electronic Supplemental Material 1

Manuscript: Full-annual demography and seasonal cycles in a resident vertebrate

Authors: Murilo Guimarães, Décio Correa, Marília Gaiarsa, Marc Kéry

Contact information: mu.guima@gmail.com, phone + 55 51 3308-7728

Abundance estimation procedure

We estimated abundance, using the parameter-expanded data augmentation (PX-DA, Royle and Dorazio 2012), where we added an arbitrary but large number of all-zero capture histories to the data (Royle and Dorazio 2012), bringing the total number of individuals in the augmented data set to size *M*. The $M - n$ augmented individuals (where *n* is the observed number of individuals), represent *potential* individuals that could be alive and within the study area. The model allows us to estimate the proportion of individuals that are actually associated with the study area. Another way to describe PX-DA in the Bayesian context is simply to note that this is a manner of specifying a discrete uniform prior between 0 and *M* for population size N (Chapter 6 in Kéry and Schaub 2012). To make sure we augmented enough pseudo-individuals, we checked the cumulative probability of inclusion, making sure its upper 95% credible interval was always below 0.7 (R. Rankin, pers. com.). Finally, we estimated recruitment probability (ψ), representing the transition from "not yet in the population" to "alive and at risk of capture". In this context, the recruitment rate includes an unobservable state and is challenging to estimate in the context of our model (Rankin et al. 2016), because the way new recruits enter the population (on either offsite or onsite states) has to be specified. Here, we set new recruits to have the same movement behavior as individuals already captured and marked in the population (Rankin et al. 2016). Due to technical reasons (Rankin et al. 2016), we constrained one of the

parameters, γ', to be constant over time, while we allowed full time-dependence for all other parameters to enable us to investigate temporal patterns in them.

Population size peaked during the first months of the breeding season (end of austral winter) when it reached 590 (CRI 390-890) individuals and was lowest in the dry/cold period, with 170 (CRI 27-400) individuals. The considerable uncertainty in the latter estimate is due to the low detection probability in that season. Females were more abundant than were males and newborns during the whole year. The number of newborns peaked in the end of hatchling period and approached zero close to the next mating season (Fig. S1).

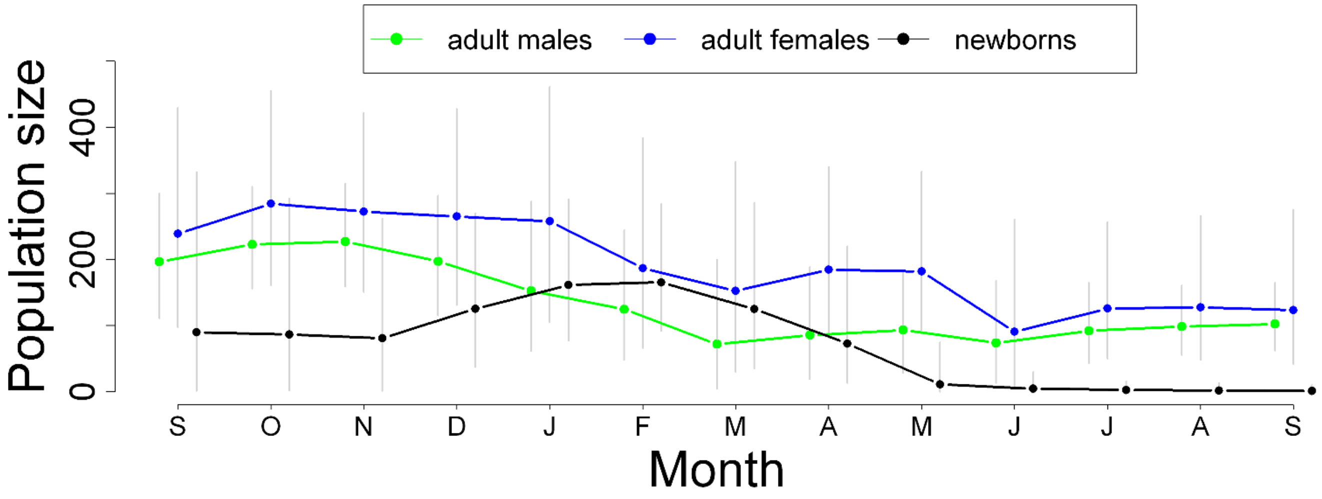

Fig. S1 Abundance estimates for adult males, adult females and newborns based on model 1 (random month effects only). Gray lines indicate 95% credible intervals
