# Supplementary material for: Full-annual demography and seasonal cycles in a resident vertebrate
Source: PeerJ. 2020 Feb 25;8:e8658. doi: 10.7717/peerj.8658 (PMC7047866; doi:10.7717/peerj.8658)
Supplement: Appendix S2 [file peerj-08-8658-s002.pdf]

Electronic Supplemental Material 2

Manuscript: Full-annual demography and seasonal cycles in a resident vertebrate

Authors: Murilo Guimarães, Décio Correa, Marília Gaiarsa, Marc Kéry

Contact information: mu.guima@gmail.com, phone + 55 51 3308-7728

R code and JAGS model

```
# This code replicates the analyses of mark-recapture data using the Robust Design adapted from Rankin
# et al. (2017) to BUGS language. Please, read Rankin et al. (2017) before running the analysis.
# This code is focused on the estimation of survival, emigration and recapture probabilities
# using a cycle predictor. Code was adapted by Marc Kéry and Murilo Guimarães


library(jagsUI)

#Load files
source("R_PCRD_JAGS_SOURCE.R") # load handy functions from Rankin et al.(2017) paper
MARK.file.name <- "RD_whiptail.inp" #load inp file

#Bundle and summarize data set
str(bdata <- list(y=Ysum, T=T, M=mm, group = group, pi = pi, month.phi = 0:11, month.p.gamma = 0:12))
#List of 7
# $ y            : num [1:1664, 1:13] 1 1 1 1 1 1 1 1 1 1 ...
# ..- attr(*, "dimnames")=List of 2
# .. ..$ : chr [1:1664] "M1" "M2" "M3" "M4" ...
# .. ..$ : NULL
# $ T            : int 13
# $ M            : int 1664
# $ group        : num [1:1664] 1 1 1 1 1 1 1 1 1 1 ...
# $ pi           : num 3.14
# $ month.phi    : int [1:12] 0 1 2 3 4 5 6 7 8 9 ...
# $ month.p.gamma: int [1:13] 0 1 2 3 4 5 6 7 8 9 ..


# Write model in BUSG language
sink("model5.txt")
cat("
    model{
    ### Priors and linear models
    # ---------------------------------------------------------------
    # Group- and time-dependent survival (interaction effects)
    # First survival is redundant and will be sampled from U(0,1)
    for(g in 1:3){
    phi[g,1] ~ dunif(0, 1)
    } # g
    # Survival for intervals 2:13 are 'real'
    for(t in 2:T){ # Loop over T=13 primary periods...
    for(g in 1:3){
    phi[g,t] <- ilogit(lphi[g,t])     #apparent survival probability on logit scale
    lphi[g,t] ~ dnorm(mu.lphi.group[g] + beta1.phi[g] * cos(2*pi*month.phi[t-1] / 12) + beta2.phi[g] *
    sin(2*pi*month.phi[t-1] / 12) , tau.lphi.time)      # Different mean, but same variance
    phi_cycle[g,t-1] <- ilogit(mu.lphi.group[g] + beta1.phi[g] * cos(2*pi*month.phi[t-1] / 12) +
    beta2.phi[g] * sin(2*pi*month.phi[t-1] / 12))
    } # g
```

```
} # t
for(g in 1:3){
mu.lphi.group[g] <- logit(mean.phi[g])
mean.phi[g] ~ dunif(0, 1)
beta1.phi[g] ~ dnorm(0, 0.1)
beta2.phi[g] ~ dnorm(0, 0.1)
}
tau.lphi.time <- pow(sd.lphi.time, -2)
sd.lphi.time ~ dt(0, 0.1, 5)I(0, )       # Half-t prior for variance (in sd scale)

# Group-dependent gamma1 constant over time
pr.gamma1 <- c(1.3,1.3)
for(g in 1:3){
gamma1[g] ~ dbeta(pr.gamma1[1],pr.gamma1[2])
} # g
# Group and time-dependent gamma2 (with interaction, and with cycles as well now)
for(t in 1:T){ # Loop over T=13 primary periods
for(g in 1:3){   # Loop over 3 groups
gamma2[g,t] <- ilogit(lgamma2[g,t]) # temp migration
lgamma2[g,t] ~ dnorm(mu.lgamma2.group[g] + beta1.gamma2[g] * cos(2*pi*month.p.gamma[t] / 12) +
 beta2.gamma2[g] * sin(2*pi*month.p.gamma[t] / 12) , tau.lgamma2.time)
gamma2_cycle[g,t] <- ilogit(mu.lgamma2.group[g] + beta1.gamma2[g] * cos(2*pi*month.p.gamma[t] / 12)
 + beta2.gamma2[g] * sin(2*pi*month.p.gamma[t] / 12))
} # g
} # t
for(g in 1:3){
mu.lgamma2.group[g] <- logit(mean.gamma2[g])
mean.gamma2[g] ~ dunif(0, 1)
beta1.gamma2[g] ~ dnorm(0, 0.1)
beta2.gamma2[g] ~ dnorm(0, 0.1)
}
tau.lgamma2.time <- pow(sd.lgamma2.time, -2)
sd.lgamma2.time ~ dt(0, 0.1, 5)I(0, )   # Half-t prior for variance (in sd scale)
# Prior for vector of probabilities for the categorical to estimate group membership
for (g in 1:3){
beta[g] ~ dgamma(1, 1)   # Induce Dirichlet prior
theta[g] <- beta[g] / sum(beta[])
} # g
# Group and time-dependent detection: (interaction effects, with cycles)
for(t in 1:T){ # Loop over T=13 primary periods...
for(g in 1:3){   # Loop over 3 groups
p[g, t] <- ilogit(lp[g, t])     # Define logit of detection probability
lp[g,t] ~ dnorm(mu.lp.group[g] + beta1.p[g] * cos(2*pi*month.p.gamma[t] / 12) + beta2.p[g] *
 sin(2*pi*month.p.gamma[t] / 12) , tau.lp.time)
p_cycle[g,t] <- ilogit(mu.lp.group[g] + beta1.p[g] * cos(2*pi*month.p.gamma[t] / 12) + beta2.p[g] *
 sin(2*pi*month.p.gamma[t] / 12))
} # g
} # t
for(g in 1:3){
mu.lp.group[g] <- logit(mean.p[g])
mean.p[g] ~ dunif(0, 1)
beta1.p[g] ~ dnorm(0, 0.1)
beta2.p[g] ~ dnorm(0, 0.1)
}
tau.lp.time <- pow(sd.lp.time, -2)
sd.lp.time ~ dt(0, 0.1, 5)I(0, )   # Half-t prior for variance (in sd scale)
# -------------------- Model for group membership ---------------------------------------------
for(i in 1:M){
group[i] ~ dcat(theta[1:3])
}
# Model for recruitment process from eigenvector decomposition
for(t in 1:T){
psi[t] ~ dunif(0,1)   # inclusion probability
}
for(t in 1:T){
for(i in 1:M){
lambda[1,i,t] <- (1-gamma1[group[i]])/(gamma2[group[i],t]- gamma1[group[i]] +1)
lambda[2,i,t] <- 1-lambda[1,i,t] # long-term prob of being outside
# trmat: transition matrix for Markovian latent-states
# 1 =not yet in population;2=dead;3=offsite;4=onsite (only observable state)
# trmat[row,column,time] = [state at time=t (arrival); state at time t-1 (departure); i #=
 individual, time=t]
trmat[1,1,i,t] <- 1-psi[t]                 # excluded from pop
trmat[2,1,i,t] <- 0                        # dead
```

```
    trmat[3,1,i,t]  <-  psi[t]*lambda[2,i,t]  #  inclusion  into  pop,  outside  study  are
    trmat[4,1,i,t]  <-  psi[t]*lambda[1,i,t]  #  inclusion  into  pop,  inside  study  area
    trmat[1,2,i,t]<- 0
    trmat[2,2,i,t]<- 1                         # stay dead
    trmat[3,2,i,t]<- 0
    trmat[4,2,i,t]<- 0
    trmat[1,3,i,t]<- 0
    trmat[2,3,i,t]<- 1-phi[group[i],t]                      # dies outside
    trmat[3,3,i,t]<- gamma1[group[i]]*phi[group[i],t]       # stays outside | outside
    trmat[4,3,i,t]<- (1-gamma1[group[i]])*phi[group[i],t]   # reenters study area | outside
    trmat[1,4,i,t]<- 0                                      # stays dead
    trmat[2,4,i,t]<- 1-phi[group[i],t]                      # dies inside
    trmat[3,4,i,t]<- gamma2[group[i],t]*phi[group[i],t]     # leaves study area | inside
    trmat[4,4,i,t]<- (1 - gamma2[group[i],t])*phi[group[i],t] # stays inside | inside
    } #i
    } # t
    # likelihood: loop through M individuals, both real and pseudo-individuals
    for (i in 1:M){
    #draw latent state at primary period 1:
    # ... by definition, everyone starts in z=1 (not-in-population) at time=0
    z[i,1] ~ dcat(trmat[1:4,1,i,1]) # first z strictly excluded from pop
    # likelihood for first primary period
    # Binomial observation process, conditional on z=4, otherwise no observation
    y[i,1] ~ dbinom(p[group[i], 1] * equals(z[i,1], 4), 7)
    # loop through primary periods after 1st primary periods
    for(t in 2:T){
    # state process: draw z(t) conditional on z(t-1)
    z[i,t] ~ dcat(trmat[1:4, z[i,t-1] , i, t])
    # likelihood
    # Binomial observation process, conditional on z=4, otherwise no observation
    y[i,t] ~ dbinom(p[group[i], t] * equals(z[i,t],4), 7)
    } # t
    } # i
    # estimate population size per primary periods
    for(t in 1:T){
    for (i in 1:M){
    # tally up alive individuals (in states 3 and 4) and those that are inside of the study area (i.e.,
   state 4)
    alive_i[i,t] <- step(z[i,t]-3) # check alive or not
    Nin_i[i,t] <- equals(z[i,t],4) # count if i is within study area
    Nin_i1[i,t] <- equals(z[i,t],4) * equals(group[i],1) # count if i is within study area
    Nin_i2[i,t] <- equals(z[i,t],4) * equals(group[i],2) # count if i is within study area
    Nin_i3[i,t] <- equals(z[i,t],4) * equals(group[i],3) # count if i is within study area
    } # i
    alive[t]  <-  sum(alive_i[,t])  #  number  alive
    Nin[t] <- sum(Nin_i[,t]) # number in study area
    Nin1[t] <- sum(Nin_i1[,t]) # number in study area and in group 1
    Nin2[t] <- sum(Nin_i2[,t]) # number in study area and in group 2
    Nin3[t] <- sum(Nin_i3[,t]) # number in study area and in group 3
    } # t
    # Calculate POPAN pent (probability of entry)
    cumprob[1] <- psi[1]
    for(t in 2:T){
    cumprob[t] <- psi[t]*prod(1-psi[1:(t-1)])
    }
    cumprob.norm <- sum(cumprob[1:T])
    # POPAN probabilities
    for(t in 1:T){
    pent[t] <- cumprob[t]/cumprob.norm
    } #t
    }", fill=TRUE)
sink()
```
